# *MTHFR* C677T Predisposes to POAG but Not to PACG in a North Indian Population: A Case Control Study

**DOI:** 10.1371/journal.pone.0103063

**Published:** 2014-07-23

**Authors:** Shashank Gupta, Pradeep Kumar Bhaskar, Ritu Bhardwaj, Abhishek Chandra, Vidya Nair Chaudhry, Prashaant Chaudhry, Akhtar Ali, Ashim Mukherjee, Mousumi Mutsuddi

**Affiliations:** 1 Department of Molecular and Human Genetics, Banaras Hindu University, Varanasi, India; 2 Department of Zoology, Banaras Hindu University, Varanasi, India; 3 Department of Ophthalmology, Institute of Medical Sciences, Banaras Hindu University, Varanasi, India; 4 R. K. Netralaya Eye Hospital & Research Centre, Varanasi, India; 5 Centre for Genetic Disorders, Banaras Hindu University, Varanasi, India; National Eye Institute, United States of America

## Abstract

Hyperhomocysteinemia induced by the C677T genetic variant in *MTHFR* (methylenetetrahydrofolate reductase) has been implicated in neuronal cell death of retinal ganglion cells (RGC), which is a characteristic feature of glaucoma. However, association of *MTHFR* C677T with glaucoma has been controversial because of inconsistent results across association studies. Association between *MTHFR* C677T and glaucoma has not been reported in Indian population. Therefore, with a focus on neurodegenerative death of RGC in glaucoma, the current study aimed to investigate association of *MTHFR* C677T with Primary Open Angle Glaucoma (POAG) and Primary Angle Closure Glaucoma (PACG) in a North Indian population. A total of 404 participants (231 patients and 173 controls) were included in this study. Genotyping was performed by Polymerase Chain Reaction-Restriction Fragment Length Polymorphism. A few random samples were also tested by direct sequencing. Genotypic and allelic distributions of the POAG and PACG cohorts were compared to that of controls by chi-square test and odds ratios were reported with 95% confidence intervals. Genotypic and allelic distributions between POAG cases and controls were significantly different (p = 0.03 and p = 0.01 respectively). Unlike POAG, we did not find significant difference in the genotypic and allelic distributions of C677T between PACG cases and controls (p>0.05). We also observed a higher proportion of TT associated POAG in females than that in males. However, this is a preliminary indication of gender specific risk of C677T that needs to be replicated in a larger cohort of males and females. The present investigation on *MTHFR* C677T and glaucoma reveals that the TT genotype and T allele of this polymorphism are significant risk factors for POAG but not for PACG in North Indian population. Ours is the first report demonstrating association of *MTHFR* C677T with POAG but not PACG in individuals from North India.

## Introduction

Glaucoma is the second most threatening eye disease attributable for blindness worldwide [1]. Therefore, understanding the genetics of glaucoma is of prime importance, not only for early detection, but also to arrest the progression of the disease. Glaucoma is a multifactorial disease, with multiple genetic and non-genetic factors contributing to its development. Myocilin (*MYOC*), Optineurin (*OPTN*), WD repeat domain 36 (*WDR36*) and *CYP1B1* are well-established causative genes for various forms of glaucoma [2], [3]. Many candidate single nucleotide polymorphisms (SNPs) have also been investigated in various populations, but known genetic factors explain only a modest fraction of glaucoma risk. Being a broad term, glaucoma encompasses clinically heterogeneous forms, all of which invariably involve a characteristic and progressive optic neuropathy leading to irreversible loss of visual field [4]. Despite this clinical heterogeneity, all forms of glaucoma ultimately result in death of retinal ganglion cells (RGC) in the optic nerve. Various molecular studies also support apoptotic death of RGC in glaucoma [5]–[7].

Hyperhomocysteinemia (hypHcy) has been shown to promote apoptosis of RGCs [8], which in turn is a result of reduction in the activity of an enzyme 5,10-methylenetetrahydofolate reductase (MTHFR, EC 1.5.1.20). MTHFR is a fundamental enzyme involved in metabolism of homocysteine (Hcy) and folate. It catalyzes reduction of 5,10-methylenetetrahydrofolate to 5-methyltetrahydrofolate, which acts as methyl donor for remethylation of Hcy to methionine. It is a crucial enzyme in this pathway that diverts folate content towards Hcy remethylation, rather than its consumption in DNA and RNA biosynthesis [9]. The C677T SNP in the *MTHFR* gene, known as rs1801133, results in an alanine to valine change at position 222 of the enzyme, which is well known to cause reduction in enzymatic activity of MTHFR and hence elevation in plasma homocysteine levels [9], [10]. In addition to various complications such as vascular injuries, neural tube defects, cardiovascular disorders and atherosclerosis, hypHcy is also known to be involved in neurodegenerative disorders [11], [12]. It is also reported to cause neuronal cell death by apoptosis or excitotoxicity [8]. In addition, elevated levels of Hcy have been reported in many forms of glaucoma [13]–[15]. Thus, taking into consideration the putative role of hypHcy in neuronal cell death and reports of hypHcy in glaucoma, an investigation of association of *MTHFR* C677T with glaucoma should enrich our knowledge about the genetic risk factors for glaucoma.

Candidate gene association studies and genome wide association studies (GWAS) have successfully identified and replicated causative genetic variants and loci for complex diseases but association of *MTHFR* C677T with glaucoma has been controversial. Association studies conducted in the populations from Europe, Japan, Sweden, Iowa (U.S.A.), Korea and Pakistan have found no association of C677T with POAG [16]–[21] whereas only one study by Junemaan et al. supports association of this polymorphism with POAG in cohorts from Germany [22]. GWAS have led to the identification of a number of susceptibility regions, such as loci near *TMCO1*, *CDKN2B* and the 9p21 locus which are associated with POAG [23]–[27]. A number of GWAS and independent case-control studies have also replicated these findings. However, there is no report of association or GWAS in POAG and *MTHFR* C677T in patients from North India. Therefore, the present study aimed to examine the genetic association of *MTHFR* C677T with two major forms of glaucoma, Primary Open Angle Glaucoma (POAG) and Primary Angle Closure Glaucoma (PACG), in a subset of the North Indian population specifically belonging to Eastern Uttar Pradesh and Western Bihar. This study is the first report demonstrating significant association of *MTHFR* C677T with open angle glaucoma but not with angle closure glaucoma in an Indian population. Our investigation also reports a higher proportion of 677TT in females affected with POAG than that in males from the same population. However, the gender specific risk of C677T towards POAG needs to be replicated in larger cohorts of both the genders. In future, it will be interesting to examine the role of *MTHFR* C677T in conferring risk of POAG in other Indian populations.

## Materials and Methods

### Subjects

In the current study, we assessed a total of 404 participants comprising 144 patients diagnosed with POAG, 87 patients with PACG, and 173 controls. All subjects were recruited from Sir Sunderlal Hospital, Banaras Hindu University, Varanasi and R.K. Netralaya Eye Hospital & Research Centre, Varanasi. Recruited participants mainly belonged to Eastern Uttar Pradesh and Western Bihar. Written informed consent was obtained from all the participants and the study was approved by the ethical committee of the Faculty of Science, Banaras Hindu University, Varanasi.

All the patients underwent a complete clinical examination by ophthalmologists for the diagnosis of POAG and PACG. Glaucoma patients with mean age of onset of approximately 60 years and fulfilling clinical criteria were included in this study. POAG was diagnosed by the following criteria: Characteristic deep glaucomatous cupping (Thin neuroretinal rim, high cup to disc ratio as compared to the size of optic disc), intraocular pressure (IOP) equal to or greater than 21 mmHg at least in one of the eyes before treatment with IOP lowering drugs, typical visual field changes of glaucoma detected by Humphrey's visual field analyzer and gonioscopically open angle of anterior chamber. 98 males (68%) and 46 females (32%) with a mean age of 59.04±10.40 years were enrolled as POAG cases. PACG was diagnosed with the same criteria as POAG except for the anterior chamber angle which is gonioscopically closed in PACG patients. The PACG cohort comprised 47 males (54%) and 40 females (46%) with a mean age of 56.31±9.13 years. Patients with systemic and/or ocular disorder in addition to glaucoma were excluded from the study. Age and ethnicity matched controls were recruited from the same hospitals. Controls were also examined by ophthalmologists to ensure complete absence of glaucomatous changes. The controls comprised 77 males (45%) and 96 females (55%) with a mean age of 60.75±9.55 years. Table 1 shows the demographic features of the study participants in detail.

**Table 1:** Demographic Features of the Study Participants.

Number of the Participants			Mean Age of the Participants			Female Participants and Mean Age			Male Participants and Mean Age		
POAG Cases	PACG Cases	Controls	POAG Cases	PACG Cases	Controls	POAG Cases	PACG Cases	Controls	POAG Cases	PACG Cases	Controls
144	87	173	59.04±10.40 years	56.31±9.13 years	60.75±9.55 years	46 (58.78±11.9 years)	40 (56.4±8.75 years)	96 (59.3±8.61 years)	98 (59.16±9.68 years)	47 (56.23±9.53 years)	77 (62.5±10.39 years)

### Molecular Analysis

5 ml of peripheral blood was collected in heparinized syringes and genomic DNA was extracted from whole blood by standard salting out procedure.

#### Genotyping of *MTHFR* C677T

**Figure 1:**
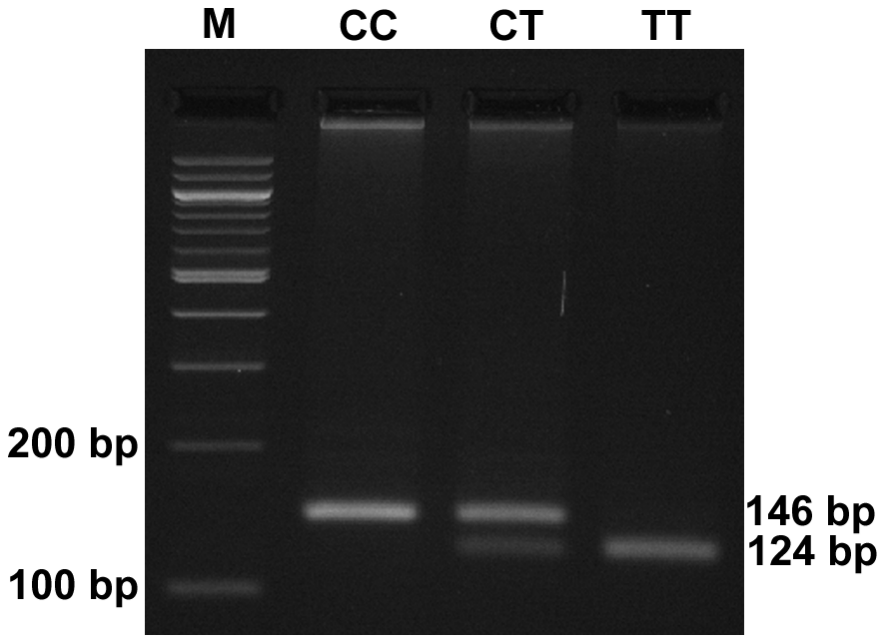
Genotyping of *MTHFR* C677T by PCR-RFLP. Substitution of cytosine (C) by thymine (T) at *MTHFR* C677T creates a restriction site for *HinfI* (5′G/ANTC3′), therefore the PCR product with CC genotype remains undigested and appears as a 146 bp band on a 4% agarose gel. The CT genotype allows the 146 bp amplicon to be digested into two fragments of 124 bp and 22 bp, and hence produces three fragments of 146 bp, 124 bp and 22 bp. However, the 4% gel cannot resolve the 22 bp fragment and only the two higher bands are visible. A single band of 124 bp is inferred as TT genotype [28]. M: 100 bp DNA Ladder (NEB).

**Figure 2:**
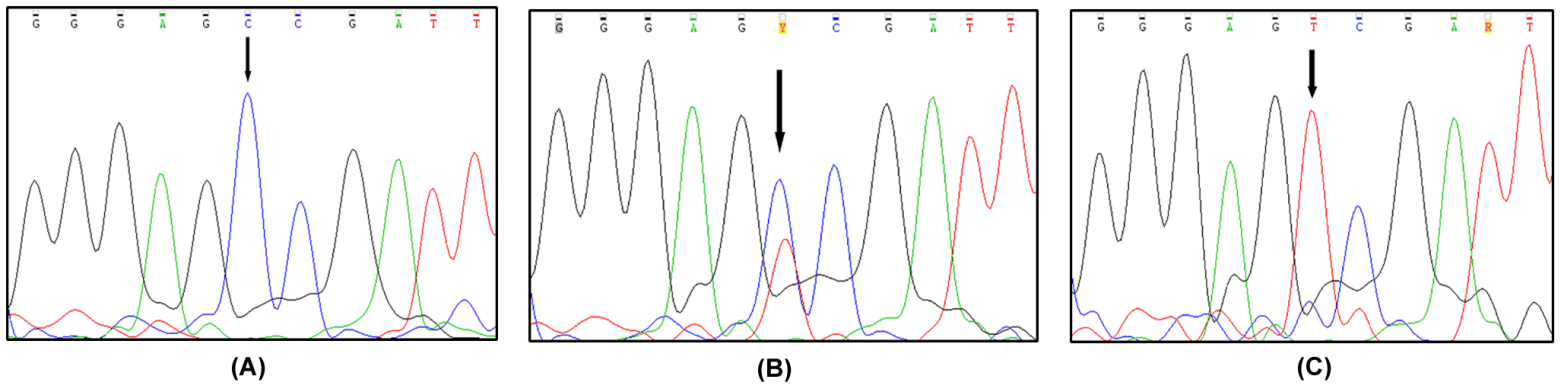
Genotyping of *MTHFR* C677T by PCR-Sanger Sequencing. Sequencing chromatograms depicting the three genotypes of *MTHFR* C677T. The SNP positions are shown by black arrows. (**A**) Homozygous wild type CC (**B**) Heterozygous CT (**C**) Homozygous mutant TT.

A fragment of 146 bp from the *MTHFR* gene containing the C677T SNP was amplified by Polymerase Chain Reaction (PCR) using forward primer (5′TGAAGGAGAAGGTGTCTGCGGGA3′) and reverse primer (5′CCTCACCTGGATGGGAAAGATCC3′) as described previously [28]. The PCR product was separated using 2% agarose gel electrophoresis to confirm the correct amplicon size. Overnight restriction digestion of the PCR product was performed at 37°C with *HinfI* enzyme (New England Biolabs, United Kingdom) according to the supplied protocol. Digestion products were resolved using 4% agarose gel electrophoresis (Figure 1). A few random samples from cases and controls were also tested by Sanger sequencing (Figure 2).

### Statistical Calculations

The genotypic frequencies of *MTHFR* C677T in the POAG, PACG, and control groups, and the total population of participants, were evaluated to satisfy Hardy-Weinberg equilibrium (p value >0.05). Genotypic and allelic distributions between cases and controls were compared for significant differences by chi square (χ2) test. Throughout analysis, p values less than 0.05 were interpreted to be statistically significant. The odds ratio (OR) was calculated to measure the risk associated with genotypes in cases with respect to controls and was reported with the 95% confidence intervals (CI).

## Results

The present study analyzed the *MTHFR* C677T polymorphism in 404 participants comprising 144 POAG cases, 87 PACG cases and 173 control participants using PCR-RFLP.

### TT genotype and T allele of *MTHFR* C677T predispose individuals toward risk of POAG

The genotypic distribution of *MTHFR* C677T in POAG cases and controls, as shown in Table 2, was found to satisfy Hardy Weinberg equilibrium. 70.14%, 24.30% and 5.56% of POAG cases had CC, CT and TT genotypes, respectively. The frequencies in POAG cases were significantly different from that of controls, where the frequencies of CC, CT and TT genotypes were 79.19%, 19.65% and 1.16%, respectively (p = 0.03, χ2 = 6.46). We also analyzed the genotypic distributions in different groups according to three different models of association studies viz. Additive Model (TT vs. CC), Dominant Model (CT+TT vs. CC) and Recessive Model (TT vs. CT+CC). POAG cases and controls exhibited significant differences in genotypic distributions according to the additive (p = 0.01, χ2 = 5.49) and recessive models (p = 0.02, χ2 = 4.97), however the p value obtained from dominant model was also very close to significance (p = 0.06, χ2 = 3.44). According to the additive model, the TT genotype conferred 4.5 times higher risk of developing POAG (OR 4.5, CI 1.2–16.2) compared to the CC genotype. Further, the recessive model showed that the TT genotype imposed 4.2 times higher risk of POAG compared to the CT and CC genotypes collectively (OR 4.2, CI 1.1–14.8). The distribution of C and T alleles in POAG cases and controls was also significantly different, as depicted in Table 2 (p = 0.01, χ2 = 5.89).

**Table 2:** Distribution of *MTHFR* C677T in POAG Cases and Controls.

Genotypic distribution					
Genotype	POAG Cases (144)	Control (173)	p (χ2)	Odds Ratio (OR)	95% Confidence Interval (CI)
**CC**	101 (70.14%)	137 (79.19%)	0.03 (6.46)		
**CT**	35 (24.30%)	34 (19.65%)			
**TT**	8 (5.56%)	2 (1.16%)			
Additive Model (TT vs. CC)			0.01 (5.49)	4.5	1.2–16.2
Dominant Model (CT+TT vs. CC)			0.06 (3.44)	1.6	0.9–2.6
Recessive Model (TT vs. CT+CC)			0.02 (4.97)	4.2	1.1–14.8

Allelic Distribution			
Alleles	POAG Cases	Control	p (χ2)
C	237 (82.29%)	308 (89.02%)	0.01 (5.89)
T	51 (17.71%)	38 (10.98%)	

### The proportion of 677TT associated POAG is higher in females

We also segregated our data on the basis of gender (Table 3), which unveiled a comparatively higher number of TT associated POAG cases in females than that in males. When we compared female patients of POAG with healthy females, we observed a significant difference (p = 0.01, χ2 = 9.18). Moreover, all three models exhibited significant differences in genotypic distributions between affected females and control females (all p values <0.05). The additive model indicated that the TT genotype in comparison to the CC genotype conferred nearly 13.5-fold higher risk of POAG in females (OR 13.5, CI 2.2–79.7). The CT and TT genotypes together were nearly 3 times more prevalent in affected females than that in control females (OR 2.7, CI 1.1–6.4). Similarly, the recessive model resulted in an OR of 11.1 with CI 1.9–63.5. The large range of confidence intervals in Table 3 might be because of low sample size after segregation on the basis of gender. Affected males did not show significant differences in genotype distributions with respect to healthy males (Table 3).

**Table 3:** Gender-wise Comparison of POAG Cases and Controls.

Females with POAG vs. Control Females				
Genotype	Females with POAG (46)	Control Females (96)	p (χ2)	OR (CI)
CC	31 (67.39%)	81 (84.38%)	0.01 (9.18)	
CT	10 (21.74%)	14 (14.58%)		
TT	05 (10.87%)	01 (1.04%)		
Additive Model (TT vs. CC)			0.003 (8.32)	13.5 (2.2–79.7)
Dominant Model (CT+TT vs. CC)			0.02 (5.38)	2.7 (1.1–6.4)
Recessive Model (TT vs. CT+CC)			0.006 (7.42)	11.1 (1.9–63.5)

Males with POAG vs. Control Males			
Genotype	Males with POAG (98)	Control Males (77)	p (χ2)
CC	70 (71.43%)	56 (72.73%)	0.74 (0.6)
CT	25 (25.51%)	20 (25.97%)	
TT	03 (3.06%)	01 (1.30%)	

When we directly compared affected females with affected males (Table 4), there was no significant difference in the genotypic distributions (p = 0.15, χ2 = 3.69). This may be because of the low sample size of affected females and males that none of the models in Table 4 showed significant difference in the genotypic distributions; however the odds ratio was more than one in each of the three cases.

**Table 4:** Comparative Genotypic Distribution of *MTHFR* C677T in Females and Males with POAG.

Genotype	Females (46)	Males (98)	p (χ2)	OR (CI)
CC	31 (67.39%)	70 (71.43%)	0.15 (3.69)	
CT	10 (21.74%)	25 (25.51%)		
TT	05 (10.87%)	03 (3.06%)		
Additive Model (TT vs. CC)			0.06 (3.39)	4.1 (0.9–19.0)
Dominant Model (CT+TT vs. CC)			0.62 (0.24)	1.2 (0.5–2.5)
Recessive Model (TT vs. CT+CC)			0.05 (3.63)	4.3 (0.9–20.1)

### *MTHFR* C677T does not exhibit statistically significant association with PACG

There was no evidence for deviation of C667T from Hardy-Weinberg Equilibrium in the PACG cohort. Among 87 PACG cases, 83.91% and 16.09% subjects were found to have CC and CT genotypes respectively, whereas none of them had the TT genotype. This genotypic distribution in cases, as shown in Table 5, did not differ significantly from that of the controls (p = 0.45, χ2 = 1.56). Table 5 also revealed no significant difference in allelic distribution (p = 0.29, χ2 = 1.10) between PACG cases and controls. Even after segregating our data on the basis of gender (Table 6 and 7), we did not observe significant differences in genotypic distributions between different pairs of cases and controls.

**Table 5:** Distribution of *MTHFR* C677T in PACG Cases and Control.

Genotypic Distribution					
Genotype	PACG Cases (87)	Control (173)	p (χ2)	Odds Ratio (OR)	95% Confidence Interval (CI)
CC	73 (83.91%)	137 (79.19%)	0.45 (1.56)		
CT	14 (16.09%)	34 (19.65%)			
TT	0 (0%)	2 (1.16%)			
Additive Model (TT vs. CC)			0.30 (1.06)	0.21	0.01–4.0
Dominant Model (CT+TT vs. CC)			0.36 (0.82)	0.73	0.3–1.4
Recessive Model (TT vs. CT+CC)			0.31 (1.01)	0.22	0.01–4.1

Allelic Distribution			
Alleles	PACG Cases	Control	p (χ2)
C	160 (91.95%)	308 (89.02%)	0.29 (1.10)
T	14 (8.05%)	38 (10.98%)	

**Table 6:** Gender-wise Comparison of PACG Cases and Control.

Females with PACG vs. Control Females			
Genotype	Females with PACG (40)	Control Females (96)	p (χ2)
CC	32 (80%)	81 (84.38%)	0.60 (0.99)
CT	8 (20%)	14 (14.58%)	
TT	0 (0%)	01 (1.04%)	

Males with PACG vs. Control Males			
Genotype	Males with PACG (47)	Control Males (77)	p (χ2)
CC	41 (87.23%)	56 (72.73%)	0.14 (3.82)
CT	6 (12.77%)	20 (25.97%)	
TT	0 (0%)	01 (1.30%)	

**Table 7:** Comparative Genotypic Distribution of *MTHFR* C677T in Females and Males with PACG.

Genotype	Females (40)	Males (47)	p (χ2)
CC	32 (80%)	41 (87.23%)	0.35 (0.83)
CT	8 (20%)	6 (12.77%)	
TT	0 (0%)	0 (0%)	

## Discussion

We initiated this study with the hypothesis that elevated levels of Hcy induced by *MTHFR* C677T could be one of the plausible factors for optic neuropathy in glaucoma, irrespective of the mechanism involved in preliminary onset of the disease. Therefore, the study aimed to explore association of *MTHFR* C677T with different cohorts of glaucoma patients.

Our study confirms significant association of *MTHFR* C677T with POAG but not with PACG in a cohort from North India, implying that the TT genotype or T allele predisposes individuals toward POAG. In the case of POAG, we also observe a higher percentage of TT in affected females than that in the affected males, which is an indication that TT might impose higher risk of POAG in females.

### Genotypic and Allelic Distribution in POAG Cohort

In our study, we observe higher frequencies of the TT genotype as well as the T allele in POAG cases than that in the controls. Our calculations based on additive model in Table 2 reveal that subjects with the TT genotype have 4.5 times higher likelihood of developing POAG than individuals with the CC genotype. The contribution of this polymorphism to POAG is again evident from the recessive model, where the TT genotype confers 4.2 times elevated risk of POAG than the CC and TT genotypes collectively. Table 2 also shows that the frequency of the T allele in POAG cases is 17.71%, whereas it is only 10.98% in controls. The frequency of the TT genotype (1.16%) and the minor allele frequency (MAF) of the T allele (10.98%) in our control group are also similar to those reported in previous studies on C677T and other disorders conducted in the same population [29]–[32]. However, the frequencies of the TT genotype and T allele are clearly higher in our POAG group than that in the controls. Our statistical findings based on various models of association studies are partially favored by a meta-analysis of POAG and *MTHFR* C677T in the year 2012 [33], which reported a significant association between the *MTHFR* C677T polymorphism and POAG in an allelic model i.e. T allele vs. C allele (OR 1.39, CI 1.05–1.83) and an additive model (OR 1.88, CI 1.04–3.43) for a population-based (PB) subgroup. This meta-analysis suggesting a strong association between the *MTHFR* C677T polymorphism and POAG in allelic and additive models for a PB subgroup indicates that the T allele or TT genotype might increase the risk of POAG, whereas no evidence of association was shown from the overall population studied.

*MTHFR* C677T has been found to be associated with elevated levels of Hcy and low levels of folate, which is different from physiological hypHcy as in fasting conditions [9]–[12]. Metabolic pathways involving Hcy remethylation require micronutrients like the vitamin B complex family, where vitamin B12 acts as a cofactor in conversion of Hcy to methionine, and folate acts as a substrate for 5-methyltetrahydrofoate. Therefore, along with genetic factors such as the T allele of the *MTHFR* C677T, nutritional status and dietary habits also modulate Hcy levels [11]. This is also evident in European and American population, where supplementation of vitamin B6, B12, and folic acid fortification counterbalance the ill effects of high T allele frequency [34], [35]. In this context, a comprehensive assessment of hypHcy and its correlation with *MTHFR* C677T in a “normal healthy” population from four eastern states of India, including subjects from Eastern Uttar Pradesh and Western Bihar, reports that the relative risk for hypHcy with 677TT is 35.5 times higher in comparison to CC and CT genotypes [29]. The study not only reconfirms the correlation between hypHcy and C677T in this population, but also suggests that dietary deficiency of vitamin B12 and folic acid can modulate the role of C677T in susceptibility to hypHcy [29]. Since the Indian population largely follows vegetarian dietary habits as compared to other populations in the world, vitamin B12 and folate levels are low here, thus nutritional status of this population further worsens the ill effects of the TT genotype.

While some studies link nutritional status, hypHcy and C677T, many other independent studies have also associated hypHcy with glaucoma. Increased plasma Hcy levels have already been correlated with the C677T polymorphism in POAG patients [13]. Higher levels of total Hcy in pseudoexfoliation glaucoma have also been demonstrated [14]. An investigation of the levels of circulating homocysteine, vitamin B6, vitamin B12 and folate in different types of open-angle glaucoma further revealed high plasma level of Hcy in pseudoexfoliation glaucoma patients along with elevated plasma levels of vitamin B6 in normal tension glaucoma and POAG sample groups [15]. In the context of our study, we focus on three interesting findings that emerge from the aforementioned studies on *MTHFR* C677T, Hcy, nutritional levels and glaucoma. The first finding associates C677T with hypHcy, the second finding highlights nutritional deficiency of vitamin B12 and folic acid in Indian populations, and the third aspect emphasizes elevated levels of Hcy in different types of open angle glaucoma. Linking these three key findings together justifies evaluating the association of C677T with POAG in this Indian subgroup.

### Gender-wise Segregation of Genotypic and Allelic Distributions

No case control association study so far has reported data on the basis of gender for *MTHFR* C677T and POAG. For the first time, we present data segregated on the basis of gender (Table 3 and 4). In terms of prevalence of glaucoma between males and females, our finding is inconsistent with a previous report where glaucoma has been shown to be more prevalent in females [1]. This could be because of the fact that studies included in the previous report were specifically conducted in a South Indian population whereas our population belongs to an entirely different subset of the Indian population. There is no comprehensive report on prevalence of glaucoma in the population examined by us. Although Table 4 shows higher incidence of POAG in males, TT homozygosity is higher in females. This indicates that there might be other genetic factors in addition to *MTHFR* C677T which have more influence in causation of POAG in males. Comparison of diseased females with control females reveals a significant difference in genotypic distribution, however direct comparison between females affected with POAG and males with POAG did not reach significant difference in the genotypic distribution. In addition, when affected males were compared with control males, significant difference in the genotypic distributions was not observed. Since, the number of females and males examined in this study is limited; it is difficult to come to a conclusion. However, the trend indicates that females are at a higher risk of TT associated POAG, where10.87% of females studied here have the TT genotype, whereas only 3.06% of affected males have this genotype (Table 4).

Association of *MTHFR* C677T and various health problems in females of this population has also been reported in other studies. An investigation of association between *MTHFR* C677T and Down's syndrome reported that young 677TT mothers had either a first or second born child with Down's syndrome in this population. Young Indian mothers with the TT genotype were reported to have genetic predisposition to non-disjunction due to abnormal folate metabolism [36]. In a different study on females from the same population, the 677T allele was found to confer significant risk toward idiopathic recurrent early pregnancy loss [37]. A plausible reason of higher risk of 677TT associated health problems in females could be relatively lower levels of vitamin B12 and folic acid than in males which augment the ill effects of the TT genotype. Thus, environmental factors such as poor nutrition, combined with genetic risk factors such as the TT genotype, could be a prominent reason for females having apparently higher risk of TT associated POAG.

### Candidate Gene and Genome Wide Association Studies

Although there are many reports available on *MTHFR* C677T and glaucoma in different ethnicities, results have been conflicting. Thus, ethnic differences appear to play a prominent role in varying genotypic and allelic frequencies that have been reported [16]–[22].

A single report so far has supported positive association of C677T with POAG in a study with 76 POAG cases and 71 cataract patients as controls [22]. Our report confirms association of *MTHFR* C677T and glaucoma from a subpopulation of North India with relatively larger sample size. A different study from central European population that assessed 204 POAG and 211 control subjects reported no association of C677T with POAG [16]. To justify this lack of association, the authors argued that there were several other non-genetic factors more important that C677T influencing Hcy levels. In a different association study of a Japanese population, association of *MTHFR* C677T was neither seen with POAG nor with Normal Tension Glaucoma (NTG). Though high frequencies of the TT genotype (20%) and the T allele (41%) were observed in the POAG group, it was not significantly different from that of controls [17]. A Swedish study involving a relatively larger sample size than ours also reported lack of association between *MTHFR* C677T and POAG [18]. Similar lack of association was also obtained in populations from Iowa (U.S.A.) and Korea [19], [20].

A separate report showed significant association of this polymorphism as well as hypHcy with PACG patients but not with POAG patients from Pakistan, specifically belonging to Punjabi ethnicity [21]. Out of 61 PACG patients recruited, 75% had CC, whereas 11% and 13% of individuals had the CT and TT genotypes, respectively. Further, they documented 1% and 13% of POAG and PACG cases with the TT genotype, respectively, which is the reverse of the frequencies we have observed in the current study. We report 5.56% of individuals with the TT genotype in the POAG group and none with TT in the PACG group. These contrasting studies may reflect the effect of ethnic differences on genotypic and allelic frequencies. Moreover, a variable frequency of T homozygosity of C677T from 1% in Africa and Southeast Asia to about 30% in Europe and America also indicates differential prevalence of this SNP in different populations [38]–[40].

GWAS has been used extensively to identify common genetic variants/loci associated with complex diseases. A few GWA studies to dissect out genetic variants associated with glaucoma have been reported in recent years [23]–[27]. These included examination of cases and controls from the United Kingdom, Japan, Australia, Canada, Netherland and a few consortia such as New Zealand Registry of Advanced Glaucoma (ANZRAG), Glaucoma Inheritance Study in Tasmania (GIST) and Wellcome Trust Case-Control Consortium 2/Blue Mountains Eye Study etc. A GWAS carried out in cases with advanced OAG from ANZRAG and the GIST identified susceptibility loci for POAG at *TMCO1* and *CDKN2B-AS* [23]. The *TMCO1* locus was replicated in a GWAS from Netherland, where a SNP located in *TMCO1* at 1q24.1 was found to be significantly associated with elevated IOP in POAG [24]. A GWAS conducted in Japanese population led to the identification of a SNP located 10 kb upstream of *CDKN2B* on chromosome 9p21 which was significantly associated with the disease [25]. Common variants in *CDKN2B-AS1* were reported to be associated with optic nerve vulnerability of glaucoma in Japanese population [26]. Association of POAG with *TMCO1* and *CDKN2B* was also supported by a GWAS from the United Kingdom [27]. In spite of these reproducible associations resulted from various GWAS and replication studies, association of *MTHFR* C677T was never reported in any whole genome association study.

Three plausible reasons can justify the absence of association between C677T and POAG in GWAS. First, *MTHFR* C677T might not be a universally prominent marker for POAG; rather it could be highly specific to North Indian population, partly because of the vegetarian dietary habit. Second, none of the GWAS reported so far has included patients from North India. Third, the phenotypic consequences of *MTHFR* C677T are significantly modulated by the dietary intake of folate and vitamin B12. Dietary deficiency of folate and vitamin B12 can further augment the severity of hypHcy caused by C677T, whereas proper dietary supplementation of the same can circumvent the ill effects of this SNP.

### Combined and Gender-wise Genotypic and Allelic Distributions in PACG Cohort

Our sample size is relatively small with only 87 cases of PACG, primarily because of lower prevalence of PACG than POAG. Still, it clearly indicates lack of association between C677T and PACG in terms of genotypic (p = 0.45, χ2 = 1.56) as well as allelic distributions (p = 0.29, χ2 = 1.10), as shown in Table 5. We observed 83.91% and 16.09% of patients with CC and CT genotype, respectively, but no patient with the TT genotype. None of the models of association studies exhibited significant differences (all p values >0.05). Therefore, it is reasonable to predict that hypHcy might not be involved in mediating neuronal cell death in PACG. Importantly, glaucoma is a complex genetic disorder. Therefore, genetic factors other than C677T might influence the pathology of angle closure glaucoma. Further, angle structure in PACG is not normal like open angle glaucoma. This abnormal narrowing of the anterior chamber angle might initiate glaucomatous events. Narrow angle hinders outflow of aqueous humor and promotes elevation in intraocular pressure (IOP), which in turn damages the optic nerve. Thus, angle mediated elevation of IOP and subsequent optic atrophy could be the primary mechanism here for causation of PACG.

In gender wise analysis (Table 6 and 7) of PACG, we compared 40 affected females with 96 control females but the genotype and allelic frequencies were not significantly different. The same was the case with 47 affected males versus 77 control males. Direct comparison of diseased females with diseased males was also not significant. Therefore, these findings suggest that *MTHFR* C677T is not associated with PACG, neither in combined population nor in any specific gender.

Our findings with respect to POAG and PACG are not surprising but rather logical. In the case of PACG, the most contrasting feature is the angle architecture that seems an obvious initiator of the disease. Additionally, we cannot deny the involvement of other genetic and non-genetic factors as well. In POAG, in spite of normal anterior angle structures, patients develop glaucomatous optic atrophy, which is an indication that there must be a combination of other genetic and non-genetic factors contributing towards optic neuropathy. The current study statistically shows that *MTHFR* C677T may be one of those contributing genetic factors.

## Conclusions

Our study is the first to investigate genetic association of *MTHFR* C677T with glaucoma in a subset of Indian population. Owing to the lack of significant association between PACG and *MTHFR* C677T, we predict that the optic neuropathy in PACG might be primarily because of abnormally high IOP that results from narrow anterior chamber angle. Moreover, glaucoma is a complex disease; therefore genetic factors other than *MTHFR* C677T could be important players in causation of PACG.

Unlike PACG, we demonstrate that the TT genotype and T allele of C677T impose increased risk for POAG in this population, supporting the hypHcy mediated death of RGC in POAG. Our study gives a preliminary indication that the proportion of TT associated POAG is higher in females than in males. However, this needs to be replicated and examined in a larger cohort of males and females affected with POAG. Having taken into account the studies conducted on modulation of Hcy levels by dietary factors, it can be suggested that the dietary supplementation of vitamin B12 and folate in individuals with the risk genotype might lessen the deleterious effects of hypHcy, and thus might lower the risk of primary open angle glaucoma. The present study not only broadens our understanding about genetic markers of glaucoma but also emphasizes the need of investigation of its association with *MTHFR* C677T in other Indian populations.
